# The Tsim Tsoum Approaches for Prevention of Cardiovascular Disease

**DOI:** 10.4061/2010/824938

**Published:** 2010-06-29

**Authors:** R. B. Singh, Fabien DeMeester, Agnieska Wilczynska

**Affiliations:** Tsim Tsoum Institute, Ul. Golebia 2, 31-007 Krakow, Poland

## Abstract

The Tsim Tsoum Concept means that humans evolved on a diet in which nature recommends to ingest fatty acids in a balanced ratio (polyunsaturated(P) : saturated(S) =w-6 : w-3 = 1 : 1)as part of dietary lipid pattern where monounsaturated fatty acids(MUFA) is the major fatty acid(P : M : S = 1 : 6 : 1) in the background of other dietary factors; antioxidants, vitamins, minerals and fiber as well as physical activity and low mental stress. Several hundred years ago, our diet included natural foods; fruits, vegetables, green vegetables, seeds, eggs and honey. Fish, and wild meat were also available to pre-agricultural humans which shaped modern human genetic nutritional requirement. Cereal grains (refined), and vegetable oils that are rich in w-6 fatty acids are relatively recent addition to the human diet that represent dramatic departure from those foods to which we are adapted. Excess of linoleic acid, trans fatty acids (TFA), saturated and total fat as well as refined starches and sugar are proinflammatory. Low dietary MUFA and n-3 fatty acids and other long chain polyunsarurated fatty acids (LCPUFA) are important in the pathogenesis of metabolic syndrome. Increased sympathetic activity with greater secretion of neurotransmitters in conjunction of underlying long chain PUFA deficiency, and excess of proinflammatory nutrients, may damage the neurons via proinflammatory cytokines, in the ventromedial hypothalamus and insulin receptors in the brain.Since, 30–50% of the fatty acids in the brain are LCPUFA, especially omega-3 fatty acids, which are incorporated in the cell membrane phospholipids, it is possible that their supplementation may be protective.Blood lipid composition does reflect one's health status: (a) circulating serum lipoproteins and their ratio provide information on their atherogenicity to blood vessels and (b) circulating plasma fatty acids, such as w-6/w-3 fatty acid ratio, give indication on proinflammatory status of blood vessels, cardiomyocytes, liver cells and neurones; (a) and (b) are phenotype-related and depend on genetic, environmental and developmental factors. As such, they appear as universal markers for holistic health and these may be important in the pathogenesis of cardiovascular diseases and cancer, which is the main consideration of Tsim Tsoum concept.

## 1. Introduction

 The epidemic of cardiovascular disease (CVD), diabetes, and cancer, in the western world and in middle income countries, indicates major increases in the incidence of generalized atherosclerosis and the total burden of CVD due to changes in the environmental factors [1–6]. Maladaptation, genetic predisposition, environmental, dietary, and lifestyle factors are the predominant contributors of CVD leading to the development of these problems [1–5]. It has been suggested that lifestyle factors such as physical inactivity and unhealthy and unbalanced nutritional consumption of excess calories, simple refined carbohydrates with a high glycemic index and load of high saturated fat (SF), high trans fatty acids (TFA), high n-6 fatty acids, and lack of monounsaturated fatty acids (MUFA) and n-3 fatty acids in the diet and reduced exercise, can cause genetic damage, contributing to the escalating rates of obesity and mortality due to CVD [3–9]. Hypertension and stroke as well as coronary artery disease (CAD), diabetes, and cancers have become a major cause of morbidity and mortality in Asia [10–13]. The obesity and CVD are contributing to major economic and public health concerns and problems that mandate aggressive, urgent, and dedicated identification, prevention, treatment, and education of public, private, and governmental agencies in both developed and middle economies [4–7].

 Adverse effects of the diet were known to Indians from the ancient times, which is evident from the following verse from an ancient scripture *Bhagwad gita.* “Foods which are bitter, acid, salted, burnt, fried, and punent, give rise to pain, mental stress, and diseases” (3100BC). Charaka (600BC), a great physician of India, knew about the role of diet and lifestyle in the pathogenesis of heart attack, which would be clear from the following verse. “Heart attack is born by the intake of fatty meals, overeating, excess of sleep, lack of exercise, and anxiety”. Charaka Sutra, 600BC. While Charak was suppose to live in Taxila University in north of India and was a Brahman physician, Sushruta was a surgeon from Vishwamitra family from Varanasi. In mahabharata, he is represented as a son of that royal sage. The garuda puranam places Divodasa as 4th in descent from Dhanavantari, the first propounder of the medical sciences upon earth, whereas the sushruta samhita describes both as same persons. Sushruta who was a surgeon, gave a more clear description of atheroscerosis or *madroga*; “Excess intake of fatty foods and lack of exercise causes obesity and narrowing of the channels taking blood to the heart. It is useful to use guggul, triphala and silajit in the treatment”. These herbs are known to have high content of antioxidant flavonoids, vitamins and minerals as well as fibres.

 In the 7th century BC, one Chinese physician proposed that “increased consumption of salt may cause hardening of the pulse”. About 2000 years ago from now (1st century), Confucius, the Chinese philosopher taught his students, “the higher the quality of foods, the better, and never rely upon the delicacy of cooking”. Thus, a dietary guideline based on experience, observation, and thinking was given as; “cereals, the basic, fruits the subsidiary, meat the beneficial, and vegetable the supplementary”. Therefore, according to WHO experts 1990, the concept of eating a diet high in animal foods and preference for meat and greasy foods was shaped in China. However, possibly the meat was rich in w-3 fatty acids, and the total fat intake remained within desirable limits and did not exceed as in the west. However, these diatary patterns were associated with enormous physical activity and sports such as hunting and also possibly meditation due to introduction of Budhism, causing possibly no significant problem of CVDs during that period. Moreover, the meat may have been from the running animals which has useful fatty acid composition. It seems that Columbus diet and lifestyle was known to Indians and Chinese from the ancient period.

## 2. Causes of Mortality

 Vascular variability disorders (VVDs) that are commonly called cardiovascular diseases (CVD) are number one causes of death globally and would be projected to remain the leading cause of death. According to WHO estimates, 17.5 million people died from VVDs in 2005, representing 30% of all global deaths. Coronary artery disease was the cause of death, among 7.6 million and 5.7 million deaths were due to stroke [1–4]. Majority of these deaths (80%) occurred in low and middle income countries. The total world population in 2007 was 6.6 billion including 1.3 billion South East Asians, which would increase to 7.9 billion and 1.6 billion respectively by the year 2025. Adult population aged 20–79 years, in the world was 4.1 billion, including 770 million South East Asians, in the year 2007, which would increase to 5.2 billion and 1083 million respectively by the year 2025. In one recent study [5], all causes of mortality were infectious diseases (41.1%, *n* = 915) such as tuberculosis, pneumonia, chronic obstructive pulmonary disease, diarrhea/dyssentary, hepatitis B, and inflammatory brain infections as the commonest cause of deaths in the urban population of north India. The second most common causes of deaths were circulatory diseases (29.1%, *n* = 646) including heart attacks (10.0%), stroke (7.8%), valvular heart disease (7.2%, *n* = 160), sudden cardiac death, and inflammatory cardiac disease, each (2.0%, *n* = 44). Malignant neoplasm (5.8%, *n* = 131), injury (14.0%, *n* = 313) including accidents, fire, and falls, and poisonings were also quite common causes of death. Miscellaneous causes of deaths were noted in 9.1%, (*n* = 202) death records, including diabetes mellitus (2.2%, 49), suicides (1.8%, *n* = 41), congenital anomalies (1.0, *n* = 37), dental caries infections (1.9, *n* = 42), and burns (1.3%, *n* = 33). Pregnancy and perinatal causes (0.72%, *n* = 15) were not commonly recorded in our study. Circulatory diseases as the cause of mortality were significantly more common among higher social classes 1–3 than in lower social classes 4 and 5 who died more often, due to infections. Heart attacks, stroke, hypertension, diabetes, and obesity were significantly more common among higher social classes 1–3 compared to class 3 and 4 but tobacco intake showed only minor differences in various classes.

 According to Registrar General of India [7], in the year 1994 to 1998, trends indicate that there has been a significant decline of proportionate deaths from infectious diseases from 22% to 16%. However, mortality from cardiovascular diseases (CVD) increased from 21% to 25% which is lower than death rate of 29.1 reported by Singh et al. in 2005 [5]. In Chennai, Gajalakshmi et al. [7] performed verbal autopsy among 48,357 adults aged 25–69 years. Deaths due to vascular diseases were 38.6% (*n* = 18, 680), followed by cancer (8.7%), tuberculosis (5.8%), and respiratory causes (3.5%). In another larger sample consisting of 150,000 subjects, there were 1,354 deaths in the first year follow up and verbal autopsy revealed that circulatory diseases as the cause of death were noted in 34% men and 30% women. Mohan et al. studied the mortality rates due to diabetes in selected urban population from south India [9] among 1399 subjects (respondents 1262). During a median follow up of 6 years, deaths were significantly greater among diabetics compared to nondiabetics (18.9 versus 5.3 per 1000 person years, *P* = .004). Mortality due to CVD were 52.9% among diabetics and 24.2 among nondiabetics. It is clear that the burden of CVD and diabetes appears to be quite significant in India indicating urgency for prevention program [4–11]. The higher risk of CAD mortality is not explained by conventional risk factors common among Indian immigrants to industrialized countries [12, 13]. Indian Society of Hypertension and International College of Nutrition and other experts have proposed guidelines for prevention of CVD and diabetes in Indians and Asians, which are being used for public education by the health workers. Social class is a determinant of mortality including cardiovascular mortality [4, 5]. It thus seems logical to examine whether differences in nutritional intake exist between the social classes, which could explain part of the existing differences in mortality due to CVD, diabetes, and cancers [4–13].

 About one fifth of the adult population in developing countries and one fourth in industrialized countries, may have CVD and diabetes [3–6]. The prevalence of diabetes and prediabetes were about 6% each in the year 2007 in South East Asia, indicating that 46.5 million subjects above 20 years had this problem which would be doubled by the year 2025. Similar targets have been calculated for CAD. The burden of hypertension would be 4-fold greater compared to diabetes. In various studies, the prevalence of hypertension (>140/90 mmHg) has been reported to be 22–30% in India among urban subjects above 20 years of age. The prevalence of CAD varies between 8% to 14% in various cities of India. In rural population, the prevalences of diabetes, hypertension, and CAD is 2-3 fold lower compared to urban subjects. Dietary intake of total fat amounts to 25%–45% in developed countries and 15%–35% of total energy in developing countries [7]. Most of the dietary fatty acids are derived from meats, oils, and dairy products, resulting in marked increase in saturated and w-6 fatty acids but relatively modest of monounsaturated fatty acids (MUFA) and long-chain polyunsaturated fatty acids (PUFAs) [10] particularly w-3 fatty acids. Refining of vegetable oils has been a major cause for increased consumption of w-6 fatty acids and hydrogenation of these oils caused greater intake of TFA, that may be the cause of mitochondrial damage in the related organs: endothelial cells, cardiomyocytes, smooth muscle cells, neurons, beta cells, and liver, causing mitochondrial damage, leading to increased prevalence of CVD, diabetes, cancers, and neurodegenerative diseases in most countries of the world [1–9]. The increase in CVD morbidity and mortality may be related to changes in diet and w-3 fatty acid consumption (Tables 1, 2, 3, 4 and 5). While Japan, *Greenland, and Mediterranean countries consuming* high w-3 fatty acid have lower CVD mortality, death rates are much higher in the northern Europe, USA, and South Asia consuming high w-6 and low w-3 diets (Tables 4 and 5).

## 3. The Evolution of Diet and the Columbus Concept

There have been marked changes in the food supply with the development of agriculture about 10,000 years ago from now. However, only nonsignificant change in our genes occurred, during the past 10 century, due to presence of w-3 fatty acids, vitamins, and minerals in the diet. The spontaneous mutation rate for nuclear DNA is estimated at 0.5% per million years. Hence, over the past 10,000 years there has been time for very little change in our genes, possibly 0.005%. Our genes appear to be similar to the genes of our ancestors during the Paleolithic period 40,000 years ago, the time when our genetic profile was established. Man appears to live in a nutritional environment which completely differs from that for which our genetic constitution was selected. However, it was only during the last 100–160 years that dietary intakes have changed significantly, causing increased intake of saturated fatty acids (SFA) and linoleic acid, and decrease in w-3 fatty acids, from grain-fed cattle, tamed at farm houses, rather than meat from running animals. There is a marked decrease in the intake of vitamins and antioxidants. The food and nutrient intake among hunter-gatherers and during Paleolithic period are given in Tables 1–3. There is a marked reduction in consumption of w-3 fatty acids, vitamins, minerals, and proteins and significant increase in the intakes of carbohydrates, (mainly refined) fat (saturated, trans fat, and linoleic acid), and salt compared to Paleolithic period.

**Figure 1:**
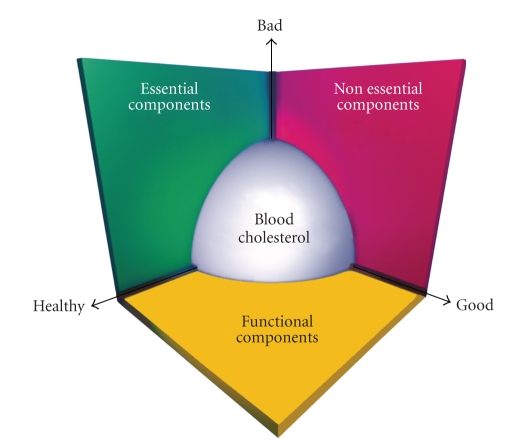
Blood cholesterol as a Marker for Holistic Health. The Columbus Concept (www.columbus-concept.com) deals with lifestyle's *essential* components to man's health. The Tsim Tsoum Concept (www.tsimtsoum.net) focuses on lifestyle's *functional* components to human health. These latter differ from the former components in that they do not contribute to the daily energy intake (DEI = ± 0) on the one hand, and tend to elevate man to human-being on the other hand. They encompass those components that characterize the noninvasive interaction of man's body/mind with his environment through recognition of his evolutionary nature, that is, heritage and development.

**Figure 2:**
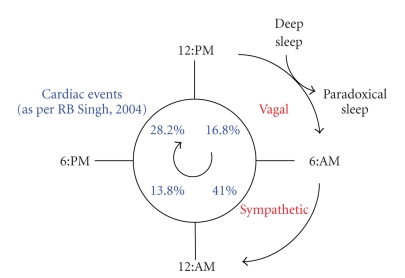
Circadian Rhythms of ACS, Columbus & Tsim Tsoum Concepts. Tsim Tsoum & Coloumbs around the clock.

 The Columbus concept of diet means that humans evolved on a diet that was low in saturated fat and the amount of w-3 and w-6 fatty acids was quite equal [15]. Nature recommends to ingest fatty acids in a balanced ratio (polyunsaturated : saturated = w-6 : w-3 = 1:1) as part of dietary lipid pattern in which monounsaturated fatty acids is the major fat (P : M : S = 1 : 6 : 1).These ratios represent the overall distribution of fats in a natural untamed environment (www.columbus-concept.com). The Columbus foods include egg, milk, meat, oil, and bread, all rich in w-3 fatty acids, similar to wild foods, consumed about 160 years ago from now. Blood lipid composition does reflect one's health status: (a) circulating serum lipoproteins and their ratio provide information on their atherogenicity to blood vessels and (b) circulating plasma fatty acids, such as w-6/w-3 fatty acid ratio, give indication on proinflammatory status of blood vessels; (a) and (b) are phenotype-related and depend on genetic, environmental, and developmental factors. As such, they appear as universal markers for holistic health. Blood cholesterol is central to this approach. Its 3D-representation shows how circulating lipoproteins affect blood vessels integrity upon their circulating throughout the body (Figure 1). Of major importance appear the essential dietary nutrients (essential amino acids, fatty acids, antioxidant vitamins, and minerals) and the functional component of the regimen (diet, sport, spiritualism, etc.). An example is given of an essential dietary nutrient and of a functional component of man's regimen that affect health in a predictive way derived from the 3D representation of blood cholesterol. Caption The Columbus Concept and its 3D representation of blood lipoprotein behaviors. “Bad” LDL-C, “good” HDLC, and “healthy” LDL-CC : HDL-CC ratios. CC = Columbus Concept. The Tsim Tsoum Concept is an extension of the Columbus concept which includes the simultaneous approach of controlling of Mind-brain-body connection by diet and lifestyle changes and by modulating mental load and circadian rhythm (Figure 2). The word Tsim Tsoum is derived from Hebreu and it is similar to ying yang in Chinese (http://www.tsimtsoum.net/editorials/tsimtsoum_editorial_2009-Kosice-14th-WCCN-and-5th-ICCD.pdf) sachs and group [16–18] used Dietary Approches to Stop Hypertension (DASH). For decreasing blood pressures, blood lipoproteins, and coronary risk. Similar dietary [19–24] interventions have been used by other workers for the last three decades to modulate blood pressure, obesity, diabetes, dyslipidemia and coronary risk in patients with high risk of CVD.These strategies appear to be similar to Columbus diet and lifestyle which may be protective, due to a favourable fatty acid ratio, antioxidants, and slowly absorbed nutrients in the diet.

## 4. Development of Cardiovascular Diseases and Diabetes

 Overweight and central obesity, may cause clustering of risk factors which may be characterized with impaired glucose tolerance, with an adverse lipid profile and oxidative stress leading to hypertension and may be seen as early as in childhood and adolescence. These risk factors, which are indicators of metabolic syndrome and CVD, tend to be clustered more rapidly in children and adolescents with unhealthy lifestyles and diets such as those with excessive intakes of saturated fats, cholesterol, refined carbohydrates and salt and inadequate consumption of dietary fibre, antioxidants, vitamins, minerals, coenzyme Q10, and w-3 fatty acids. Low power mitochondria, due to coenzyme Q deficiency, may be associated with reduced aerobic capacity and predispose the metabolic syndrome and CVD [25–30]. Lack of physical activity and increase in television viewing are other factors which further increase the risk by decreasing basal metabolic rate (BMR) [1–3]. In older children and adolescents, habitual alcohol and tobacco use also contribute to high blood pressure and to the development of other risk factors in early adulthood which continue to act in later life course. Such clustering of risk factors which is characteristic of metabolic syndrome and CVD, represents an opportunity to address more than one risk factor at a time and may be due to clustering of health related behaviours [30]. Of the several characteristics of metabolic syndrome, at least 3 should be present for its diagnosis. Obesity in conjunction with type 2 diabetes, hypertension, coronary artery disease (CAD), and dyslipidemia are important features of the metabolic syndrome which is usually associated with hyperinsulinemia and insulin resistance [25, 26, 31].

 There is coexistence of nutritional deficiencies and appreciable overnutrition in the form of central obesity and overweight in both developed and developing countries [11–13, 15–37]. Cohort studies clearly showed that the gratifying gains in cardiovascular health occurred in developed countries, in association with an epidemic of CVD in the developing world [25–27, 31]. We have been in a position to learn, the mechanism of transition from poverty to economic development and emergence of cardiovascular disease (CVD) [11, 32]. It seems that metabolic syndrome is an important pathway for development of CVD and type 2 diabetes. We proposed that “overweight comes first in conjunction with hyperinsulinemia, increased angiotensin activity, increased proinflammatory cytokines and central obesity followed by glucose intolerance, type 2 diabetes, hypertension, low high density lipoprotein cholesterol (HDL) and hypertriglyceridemia (Metabolic syndrome) [25–27]. This sequence is followed by CAD, gall stones and cancers and finally dental caries, gastrointestinal diseases and bone and joint diseases, during transition from poverty to affluence”. As people become rich, they begin to increase their dietary fat, salt and sugar (proinflammatory foods) intake in the form of ready prepared foods, syrups, dairy products and flesh foods in place of grain based diet [27]. There is a greater use of automobiles, television viewing and decrease in sports, walking and dancing as recreation. These changes in the diet and lifestyle in conjunction with increased tobacco and alcohol intake, appear to be basic factors in the pathogenesis of noncommunicable diseases, including CVD [3, 31]. The last few decades of the 20th century offered us an opportunity to initiate action to counter growing epidemics of CVD and type 2 diabetes, on both sides of the Atlantic [25–30, 38]. When people learned the methods of prevention, there was a decrease in CVD in the western world but obesity continued to increase, resulting into an increase in the metabolic syndrome in both developed and developing economies [25, 26, 31]. It is possible that these adverse diet and lifestyle factors damage the mitochondria of the cardiovascular system and ventromedial hypothalamus, leading to CVD and diabetes.

## 5. Fatty Acids in the Diet and Development of CVD and Diabetes

 There has been an enormous increase in w-6 fatty acid (about 30 g/day) in the diet due to the production of oils from vegetable seeds such as corn, sunflower, saiflower, soybean, and cotton. Increased intake of meat has resulted into greater intake of arachidonic acid (0.2–1.0 mg/day), whereas the consumption of alpha-linolenic acid (ALA) has decreased (about 0.55 g/day) and the amounts of eicosapentaenoic acid (EPA) and docosahexaenoic acid (DHA) are 48 and 72 mg/day, respectively, [25–29, 31, 37]. A relative and absolute decrease in w-3 fatty acids has led to an imbalance and increase in the ratio of w-6/w-3 fatty acids to upto 50 in India and other developing countries, consuming vegetable seed oils (corn, soyabean, sunflower, and cotton) [27–30, 38–40]. Saturated fatty acids (SFA) and trans fatty acids (TFA) elevate, PUFA decrease and MUFA have beneficial effects on total and low density lipoprotein cholesterol (LDL) as well as on HDL cholesterol. Omega-6 PUFA and TFA also decrease HDL cholesterol, and increase insulin resistance, free radical stress, and inflammation, which may enhance atherosclerosis [27–29]. Increased intake of total fat, TFA, SFA, and w-6 fatty acids and refined carbohydrates, may cause insulin resistance resulting into metabolic syndrome [23, 24, 31–37]. Decreased intake of MUFA, w-3 fatty acids, fiber, and phytochemicals may enhance the metabolic syndrome [15–37].

## 6. Cardiovascular Disease in Developing Countries

 Coronary risk factors in the developing world include low concentration of HDL cholesterol, hyper triglyceridemia, abdominal obesity, high prevalence of type 2 diabetes and CAD, and hypertension, indicating presence of metabolic syndrome and CVD [38–41]. Sedentary lifestyle, increase in dietary fat, refined carbohydrate, and alcohol consumption are common behaviour patterns underlying above risk factors. Insulin resistance is nearly universal in all these conditions and South Asians are at the greatest risk of developing CAD and diabetes [30, 38–40]. These behaviour patterns result into hyper insulinemia but insulin resistance may differ and may not occur in all tissues of the body. Adipose tissue is not resistant to insulin in the early stages of whole-body insulin resistance, but muscle is resistant very early in the progression of metabolic syndrome X. Therefore, physical activity and yogasans, appear to be important in the prevention and treatment of insulin resistance. There is no scientific evidence to demonstrate that metabolic syndrome among South Asians is genetic in origin. It is possible that populations in developing countries, under scarcity, may have adapted to survive at low fat intake and physically demanding occupations, which made them more susceptible to dietary energy, sedentary behavior, and to established risk factors [42–50]. Abdominal obesity (central deposition of fat) in South Asians, appears to be universal in all the countries, wherever they are living, but whether the development of this type of obesity is genetic, dietary, caused by low physical activity, or a combination of these factors has not been established. Central obesity appears to be more prominent in the South Asian population than in the other Asian and Western populations. It is possible that transgenic mice overexpressing 11*β* hydroxysteroid dehydrogenase type 1 (11*β*HSD-1) selectively in adipose tissue can develop abdominal obesity and exhibit insulin-resistant diabetes, hyperlipidemia, and hyperphagia despite hyperleptinemia [50]. It is not known whether adipose tissue from the abdomens of South Asians show increased 11*β*HSD-1 activity. If this is true, peroxisome proliferator-activated receptor-*γ* ligands, which markedly reduce adipocyte 11*β*HSD-1 activity in vitro and in vivo preferentially reduce abdominal fat, may be the drugs of choice in South Asians to reduce insulin resistance and obesity [43, 50]. South Asians may be genetically programmed or due to maladaptation may overxpress 11*β*HSD-1 in their adipose tissue, which may account for their higher risk of metabolic syndrome. It is possible, that treatment of central obesity with these agents, may be protective against metabolic syndrome, in South Asians [43, 50].

 Singh et al. [43], in one cross-sectional survey, among 255 rural 311 urban elderly subjects, showed that mean blood pressures, body mass index, insulin levels, and the prevalence of hypertension were significantly greater among urban compared to rural population. Total fat intake was significantly greater among urban and hypertensive subjects compared to rural normotensive and hypertensive subjects, respectively. A recent study [46], among 54 patients of acute myocardial infarction (AMI), showed that the intake of large meals and large breakfast >1000 cal especially rich in TFA was significantly associated with AMI compared to control subjects. Those consuming large meals, showed significantly greater levels of tumour necrosis factor-alpha (TNF-*α*) and interleukin-6 (IL-6) compared to subjects taking small breakfast. In another study [34], among 202 patients, large meals was a trigger for development of AMI among half of the patients. TNF-*α* and IL-6, incidence of known hypertension, and type-2 diabetes were significantly greater among AMI patients compared to healthy subjects. These proinflammatory markers are risk factors of AMI and metabolic syndrome [42]. In one cross-sectional survey [47], among 3257 women, aged 25–64 years, social class 1–3 were consuming significantly greater amount of total visible fat, including TFA, clarified butter (Indian ghee), and vegetable oils, compared to lower social classes 4 and 5. Mean body mass index (BMI), over weight (BMI>25 Kg/M2), and central obesity (Waist-hip ratio> 85) were significantly greater among higher social classes than lower social classes.

 Higher social classes 1-3 are known to have greater prevalence of CAD, type 2 diabetes and hypertension, indicating metabolic syndrome in Indians. It is possible that lower intake of w-3 fatty acids and MUFA, as well as increased consumption of w-6 fatty acids, SFA, and TFA may be responsible for central obesity and metabolic syndrome among these patients [40, 47–49]. In another study [50], among 850 men, aged 25–64 years, subjects were divided into high fat, over-fat, normal-fat, and under-fat based on criteria of body fat analysis by bioelectrical impedance. The prevalence of CAD, type 2 diabetes, and hypertension, as well as low HDL cholesterol, BMI, and WHR were significantly associated with high body fat percentage.

## 7. Interactions of Gene and Environment

 Dietary factors, physical activity, mental stress, and environmental toxicants can influence gene expression and have shaped the genome over several million years of human evolution [14, 44, 49] which has given an opportunity for health, as well as susceptibility to diseases, through genes, while environmental factors determine which susceptible individuals will develop metabolic syndrome. Rapid changes in diet and lifestyle due to socioeconomic changes provide added stress causing exposure of underlying genetic predisposition to chronic diseases such as type 2 diabetes, obesity, hypertension, CAD, and atherosclerosis. Several studies are continuing on the role of nutrients in gene expression [49]. It is not clear how n-3 fatty acids suppress or decrease the mRNA of interleukin, which is elevated in atherosclerosis, arthritis and other autoimmune diseases, whereas n-6 fatty acids have no such effects [49]. Metabolic syndrome appears to be polygenic in nature and rapidly escalating rates suggest the importance of environmental change, rather than changes in genetic susceptibility.

 It has been proposed [42–50] that genetic and other factors, including oxidative stress; superoxide anion (O_2_) and hydrogen peroxide (H_2_O_2_), endothelial nitric oxide (eNO), lipid peroxides, antioxidants, endothelin, angiotensin converting enzyme (ACE) activity, angiotensin-II, transforming growth factor-*β* (TGF-*β*), insulin, homocysteine, asymmetrical dimethyl arginine, proinflammatory cytokines: interleukin-6 (IL-6), tumor necrosis factor-*α* (TNF-*α*), C-reactive protein (hs-CRP), long-chain polyunsaturated fatty acids (LCPUFAs), and activity of NAD(P)H oxidase have a role in human essential hypertension. There is a close interaction between endogenous molecules: (endothelial nitric oxide) eNO, endothelin, cytokines, and nutrients: folic acid, coenzyme Q10, L-carnitine, L-arginine, tetrahydrobiopterin (H_4_B), vitamin B6, vitamin B12, vitamin C, and LCPUFAs. Statins can mediate some of their actions through (LCPUFAs), whereas these fatty acids (especially *ω*-3 fatty acids) suppress cyclooxygenase activity and the synthesis of proinflammatory cytokines, and activate parasympathetic nervous system. This activity can reduce the risk of major vascular events. LCPUFAs such as EPA, DHA from precursors to lipoxins and resolvins may have antiinflammatory actions. Low-grade systemic inflammation seen in hypertension seems to have its origins in the perinatal period and availability of adequate amounts of LCPUFAs during the critical periods of brain growth prevents the development of hypertension [14]. This indicates that preventive strategies aimed at decreasing the incidence of hypertension and its associated conditions such as atherosclerosis, type 2 diabetes, CAD, and cardiac failure in adulthood need to be prevented during the perinatal period for primordial prevention of metabolic syndrome.

## 8. Dietary Factors and Cardiovascular Disease

 Experimental studies indicate that type and amount of dietary fatty acids may cause insulin resistance and metabolic syndrome [51]. MUFA or w-3 fatty acids appear to have beneficial effects on insulin action, whereas w-6 fatty acids, saturated fats and diets with high total-fat content, appear to decrease insulin sensitivity in animal studies [30, 38, 39]. It is hypothesized that dietary fats affect the phospholipid composition of cell membranes in skeletal muscle and other tissues. Several clinical studies showed a decrease in insulin sensitivity with high fat diets [52, 53]. A few studies diminish the strength of their conclusions, including large difference in diets, the nonrandomized assignment of diets, and lack of standardized methods to measure insulin sensitivity. A few studies using more standard measures reported a relationship between fat content and insulin sensitivity [54]. One reason may be the relatively short duration of intervention in many of these studies. A recent multicenter, 3-month investigation found that a diet high in saturated fat (18% of energy) decreased insulin sensitivity more than a diet high in monounsaturated fat (21% of energy) among 162 healthy men and women [55]. Many cross-sectional epidemiologic studies also demonstrated positive association between intake of saturated fat and hyperinsulinemia, after adjustment for measure of body fat [56, 57], but at least one large, well-designed study showed no association [58]. Prospective studies including the Nurses' Health Study [59] suggest the role of specific types of fat in the development of type 2 diabetes mellitus. In the Nurses' Health Study, investigators reported an inverse association between development of diabetes and intake of vegetable fat and polyunsaturated fat, a positive association for trans-fatty acids, but no association for total fat in the diet. In a more recent study [60], among 15 obese, hyper insulinemic subjects, a hypoenergetic, MUFA-rich diet containing 35% carbohydrate, 45% fat, and 20% protein was compared with a diet containing; 60% carbohydrate, 20% fat, and 20% protein. After 4 weeks, fasting insulin levels, insulin to glucose ratio, Homeostatic Model, and Assessment Index decreased to normal ranges and were significantly lower in high MUFA group as compared with the control group. However, insulin sensitivity score increased significantly, waist circumference showed significant decline, in the high MUFA group compared to high carbohydrate group, indicating improved insulin sensitivity and decreased central obesity, respectively, with MUFA-rich diet. In a cross-sectional population study [61], Folsom et al. reported among 4000 healthy subjects that fasting insulin levels were positively associated with the percentage of saturated fat in plasma, and inversely associated with percentage of MUFA. Lovejoy et al. [62] observed that certain class of fatty acids such as w-6, saturated and TFA have more deleterious effects on insulin action than others, and increase the risk of type 2 diabetes mellitus and therefore metabolic syndrome. Low and coworkers [63] found that a high MUFA diet induced improvement in the control of type 2 diabetes as compared to high carbohydrate diet. Similar results were noted by other workers [60, 64]. In one study, MUFA-rich diet also reduced the total cholesterol to HDL-cholesterol ratio, compared to high carbohydrate diet [65]. 

 A greater reduction in triglyceride levels was observed in the high MUFA group than in the high carbohydrate group [65, 66]. These results were observed in earlier studies [67] showing that dietary fatty acid composition affects the fatty acid composition of VLDL-triglyceride and alterations in composition of VLDL and in the enzymes involved in its catabolism are two mechanisms observed in the hypotriglyceridemic effect of high MUFA diets [68]. Oleic acid has also been found to cause a reduction in triglyceride levels, possibly through increasing the removal of triacylglycerol [69]. Moreover, olive oil has been found to promote gastrointestinal secretions and stimulate stomach emptying, thereby increasing the rate of supply of fatty acids to the enterocytes [70], thus accelerating the rate of digestion and absorption and faster rate of entry of chylomicrons into the circulation. This implies that the long-term use of olive oil may cause up-regulation of the enterocytes' ability to process dietary triacylglycerol and synthesize chylomicrons [71]. However, high carbohydrate diets are known for their hypertriglyceridemic effects and glucose intolerance [72] which appear to be due to downregulation of muscle lipoprotein lipase (LPL) activity [73]. These adverse effects of such diets can enhance diabetes and cardiovascular disease or metabolic syndrome [74–78]. The Columbus concept of diet; including fruits, vegetables, whole grains, nuts, w-3 fatty acid rich egg, and meats, and Columbus oil (olive oil+lin seed oil) in conjunction with physical activity addresses both diet and lifestyle, may be useful in the prevention of metabolic syndrome as well as its components [14, 79–81].

## 9. Diet and Inflammation and Endothelial Dysfunction

Vascular indexes should be independently considered as risk factors of atherothrombosis, because of antiinflammatory effects of statins, hormone replacement therapy (HRT) and postprandial endothelial dysfunction, in relation to inflammation [27–29] impaired vascular biology, physiology, and biochemistry resulting into inflammation and endothelial dysfunction may be independently atherothrombotic [27, 82].

 There is evidence that abnormalities of the postprandial state are important contributing factors to the development of atherosclerosis [45, 46, 82]. Recent studies indicate that changes in LDL cholesterol and C-reactive protein independently correlated with coronary atherosclerosis progression and coronary events [83, 84]. However, on treatment, C-reactive protein was as predictive of subsequent coronary events as was LDL cholesterol. HRT increases HDL cholesterol, and endothelial function, as well as inflammation and coagulation which are atherogenic, hence HRD has been discarded.

 Clinical data indicate that postprandial hypertriglyceridaemia is a risk factor for cardiovascular disease in non-diabetic subjects and may be a predictor of carotid intima-media thickness in type 2 diabetic patients [85]. Meal absorption is a complex phenomenon, and postprandial hyperlipidaemia and hyperglycaemia are simultaneously present in the postabsorptive phase, particularly in diabetic patients or in subjects with impaired glucose tolerance [9, 82, 85, 86]. Both postprandial hyperglycaemia and hypertriglyceridaemia may cause endothelial dyfunction, which is considered an early marker of atherosclerosis [27]. Effect of different isocaloric meals on endothelial function in both normal subjects and type 2 diabetic patients, may be that the level of triglycerides after a high-fat (saturated) meal may be associated with endothelial dysfunction, with maximal impairment occurring at the time of the simultaneous presence of postprandial hyperglycaemia and hypertriglyceridaemia [27]. The effect of liquid meals rich in carbohydrates or saturated fats may be similar. It is possible that endothelial dysfunction induced by a high-fat meal in type 2 diabetic patients or associated with fasting hypertriglyceridaemia in young men could be associated with increased plasma concentrations of asymmetric dimethylarginine, an endogenous nitric oxide synthase inhibitor, suggested as a novel cardiovascular risk factor [87].

A mild prooxidative state accompanies meal ingestion, which results in raised circulating biomarkers of inflammation, adhesion, and endothelial dysfunction, all of which are factors in the development of cardiovascular disease [88]. The effect of hyperglicaemia, hypertriglyceridaemia, and raised free fatty acids (FFA) levels both fasting and postprandial, on endothelial function may be mediated through the generation of an oxidative stress [27, 88]. The process is supposed to involve increased superoxide generation, which in turn inactivates nitric oxide. Superoxide and nitric oxide combine to produce peroxynitrite, a potent and long-lived oxidant, that is, cytotoxic, initiates lipid peroxidation and nitrates amino acids such as tyrosine which affects many signal transduction pathways. The production of the peroxynitrite anion can be indirectly inferred by the presence of nitrotyrosine (NT). An increase in plasma NT levels has been reported in association with postprandial hyperglycaemia or hypertrigly-cidaemia, with a cumulative effect occurring in the presence of both conditions [27–29]. It seems therefore that oxidative stress is a mediator of the effect of raised substrate concentration in the postprandial phase [89, 90]. It is clear that what happens during the absorption phase may be of considerable importance, because it occurs several times every day and human beings now spend an increasingly greater part of their lives in the postprandial phase without periods of fasting. These biological markers after a high-fat meal, also rich in refined carbohydrates, appear to be basic underlying mechanism for insulin resistance and metabolic syndrome leading to CVD [91].

## 10. Proinflammatory Macronutrients

 Proinflammatory macronutrients such as w-6 fatty acids, TFA and SFA as well as refined carbohydrates intake may produce oxidative stress and proinflammatory substances [27] (Figures 1 and 2). Glucose ingestion in normal subjects is associated with increased superoxide generation in leukocytes and mononuclear cells, as well as with raised amount and activity of nuclear factor-*κ*B (NF-*κ*B), a transcriptional factor regulating the activity of at least 125 genes, most of which are proinflammatory [91]. Increased consumption of refined carbohydrates also causes an increase in two other proinflammatory transcription factor, activating protein-1 (AP-1), and Egr-1, the first regulating the transcription of matrix metalloproteinases and the second modulating the transcription of tissue factor and plasminogen activator inhibitor-1 [27, 91]. A mixed meal from a fast-food chain has also been shown to induce activation of NF-*κ*B associated with the generation of reactive oxygen species (ROS) by mononuclear cells. Superoxide anion appear to be an activator of at least two major proinflammatory transcription factor, NF-*κ*B and AP-1. These observations are consistent with previous findings, demonstrating that after oral or intravenous glucose challenges, in both normal subjects and patients with type 2 diabetes mellitus, there is an increased generation of ROS and raised circulating levels of proinflammatory cytokines, such as TNF-*α*, IL-6, and IL-18 [27, 92–94]. In apparently healthy subjects, a single high-fat meal produces endothelial activation, as evidenced by increased concentrations of the adhesion molecules VCAM-1 (vascular cell adhesion molecule-1) and ICAM-1 (intercellular adhesion molecule-1), in association with raised plasma concentrations of IL-6 and TNF-*α* [27]. A high-fat meal [94] may increase the circulating levels of IL-18, a proinflammatory cytokine supposed to be involved in plaque destabilization associated with the simultaneous decrease of circulating adiponectin, an adipocyte-derived protein with insulin sensitizing, antiinflammatory, and antiatherogenic properties [95].

## 11. Diet, and Insulin Resistance

 Biological dysfunctions, found in diabetes, obesity, and the metabolic syndrome include, among others, increases in the circulating levels of metabolites, such as FFA and triglycerides, and cytokines such as TNF-*α* and IL-6. Administration of a marcronutrient, causes a shift towards oxidative stress and inflammation, which in turn may reduce insulin sensitivity. FFA as well as proinflammatory markers have been shown to predict type 2 diabetes independent of known risk factor [96, 97]. Both FFA and TNF-*α* have also been shown to activate inhibitor K kinase *β* (IKK-*β*) in adipocytes and hepatocytes, which can then increase the serine phosphorylation of insulin receptor substrate1 (IRS-1), with subsequent reduction in insulin-dependent tyrosine phosphorylation of IRS-1, and ultimately glucose transport [98]. IKK*β* is a serine kinase that controls the activation of NK-*κ*B, a transcription factor associated with inflammation. IRS-1 may be directly phosphorylated by IKK-*β* at serine residues, respresenting a novel class of substrates for IKK*β* [99]. In one recent study [100] in which hepatic expression of the lkappaBalpha superrepressor, which reduces IKK*β* activity, reversed the phenotype of wild-type mice fed diet. It is possible that lipid accumulation in the liver leads to subacute hepatic “inflammation” through NK-*κ*B activation and downstream cytokine production resulting into insulin resistance both locally in liver as well as systemically. Circulation of IL-6 in plasma is at high concentrations and is associated with insulin resistance in men and in obese or hyperandrogenic women [27]. Circulating IL-6 levels and insulin sensitivity relationships seem to occur in parallel to increases in plasma FFA. In contrast to IL-6 and TNF-*α*, adiponectin mRNA is reduced in adipose tissue from patients with type 2 diabetes [95]. It seems that low adiponectin production contributes to insulin resistance and there is evidence that adiponectin decreases circulating FFA levels by increasing fatty acid oxidation by skeletal muscle [101]. The endogenous proinflammatory potential may be greater, in the postprandial phase, due to imbalance in pro and antiinflammatory cytokines, particularly following the ingestion of rapidly absorbed foods. Modification of circulating FFA levels may mediate a part of this effect and blunting of antiinflammatory actions of insulin might also play a role. Insulin causes a suppression of NK-*κ*B, at physiologically relevant concentrations, thus reducing the production of some of its transcripts, namely IL-6 and TNF-*α* [27]. This benefit of insulin, has been related to its ability to induce the release of nitric oxide and to enhance the expression of constitutive nitric oxide synthase.

## 12. Dietary Approaches to Stop Endothelial Damage

 The vascular effects of high sugar and high fat meals have greatly increased our understanding about the role of diet on atherothrombosis [28]. There is increased flux of nutrients in the postprandial state which is associated with an increase in circulating levels of proinflammatory cytokines, recruitment of netrophils and oxidative stress. The generation of reactive oxygen species (ROS) may be a common ground for all these findings and may help understanding current dietetic recommendations of the International College of Cardiology, emphasizing increased consumption of fruits, vegetables, (400 g/day), nuts (50 g/day), and grains (400 g/day), spices, and MUFA+w-3 fatty acids rich oils (30–50 g/day). Fruits, vegetables, nuts and spices are rich in natural antioxidants, phytochemicals, and fibre that help fighting the oxidant wave of meals. Decreasing the intake of w-6, trans- and saturated fatty acids and increasing the consumption of omega-3 fatty acids (lin, mustered, and canola oil) and MUFA (olive oil) are also considered important strategies to reduce CAD and metabolic syndrome [25, 26, 31].There is evidence that these two strategies are also associated with a reduced inflammatory status. In the Nurses Health Study, levels of C-reactive protein and markers of endothelial dysfunction were 73% higher in the highest quintile of trans-fatty acids intake, compared with the lowest quintile [102] and low-cholesterol/low-saturated fat diets are associated with mitigation of low-grade systemic inflammation which correlated with reduction of plasma C-reactive protein levels [103]. Cross-sectional study from the Nurses' Health Study I cohort demonstrated lower concentrations of many markers of inflammation and endothelial activation, including C-reactive protein, IL-6, and E-selectin, among those in the highest quintile of omega-3 fatty acids, when compared with the lowest quintile [104]. Since a high intake of omega-6 fatty acids may reduce the known beneficial effects of omega-3 fatty acids on CAD risk [49]; a combination of both types of fatty acids in a ratio of 1 : 1 as advised by Columbus concept Institute, which may be associated with the lowest level of inflammation [105].

However, it seems that w-6/w-3 ratio in the diet should be <5.0 to have optimal benefit of these fatty acids in the prevention of CVD, type 2 diabetes, and metabolic syndrome.

 Since free radical stress is supposed to play a key role in the development of atherosclerosis, antioxidant-vitamin supplementation has been suggested for the treatment and prevention of chronic diseases, including CAD [44]. The encouraging results of short-term trials in participants with coronary atherosclerosis were not confirmed in large-size intervention trials. It seems that it is wrong to focus on a single element of the diet; guideline from some professional or governmental panels recommend to consume vitamins and minerals from food sources, rather than from supplements [44, 105]. A shift towards energy dense, refined, ready prepared foods with high glycemic index (refined starches and sugar) and unhealthy lipids (TFA, SF, and w-6 rich oils) poor in phytochemicals and fibre have been adopted by increased number of people and populations in the western world and in the urban populations of middle income countries in the last few decades [26, 42–48]. These changes in diet can cause an inctivation of innate immune system, by excessive production of proinflammatory cytokines and reduced production of antiinflammatory cytokines, which may result into generation of inflammatory milieu, causing insulin resistance, and endothelial dysfunction. These changes in diet in conjunction with inadequate physical activity appear to be responsible for the development of positive energy balance, weight gain, and central obesity, which is widely acknowledged as an endocrine organ, secreting an increasing number of mediators, including proinflammatory cytokines [106]. Central obesity is a key promoter of low grade systemic inflammatory state [85, 86] and is characterized by the most severe metabolic abnormalities [28]. It seems that subjects with abdominal adiposity are particularly prone to the proinflammatory effects of unhealthy diets. The changes in dietary patterns that occurred in recent years are characterised with the intake of large amount of foods that seem faster in preparations and producing health damage. One prospective CARDIA (Coronary artery risk development in young adults) study indicate that frequent fast food intake causes weight gain and risk of insulin resistance, over 15 years [107].

 The Quebec Family Study has shown that a decrease in the consumption of fat-foods or an increase in consumption of whole grains and fruits predicted a lower increase in body weight and adiposity indicators, over a 6-year follow-up [108]. However, no specific dietary recommendations have been advocated by health agencies for the treatment of insulin resistance or the metabolic syndrome [44, 80, 81]. Given that the metabolic syndrome is an identifiable and potentially modifiable risk state for both type 2 diabetes and cardiovascular disease, adopting a dietary pattern as that used by other workers may reduce the potential risk of these diseases [44, 109–113]. In one study, Knoops et al. [114], (Colleagues from the Netherlands, France, Spain and Italy) have demonstrated that in European men and women aged 70–90, adherence to a Mediterranean-style diet, which represents a solid example of a healthy dietary pattern, moderate alcohol consumption, nonsmoking status, and physical activity was associated with a lower rate of all-cause mortality. This combination of healthy diet and lifestyle, was associated with a mortality rate of about one-third that of those with none or only one of these protective factors. Another larger study, involving about 22 000 adults, showed an inverse correlation between a greater adherence to a Mediterranean-style diet and death [115]. Approximately a 2/9 increment in the Mediterranean diet score was associated with a 25% reduction in total mortality and a 33% reduction in CAD mortality.

## 13. Intervention Trials

 Intervention trials, using the whole diet approach so far produced are also in line with this epidemiological evidence. In the Lyon Diet Heart Study [116], 605 patients who had a myocardial infarction were randomly assigned to a “Mediterranean-style” diet or a control diet resempling the American Heart Association Step I diet. The Mediterranean diet model supplied 30% of energy from fats and <10% of energy from saturated fatty acids, whereas the intake of 18 : 3 (*n*-3) (*α*-linolenic acid) provided >0.6% of energy. After a mean follow-up of 27 months, the risk of new acute myocardial infarction and episodes of unstable angina was reduced by ~70% by the Mediterranean diet. Moreover, total mortality was also reduced by 70%. Singh et al. [109] tested an “Indo-Mediterranean diet” in 1000 patients in India, with existing coronary disease or at high risk for coronary disease. When compared with the control diet, the intervention diet characterized by increased intake of mustard or soyabean oil, nuts (walnuts, almonds), vegetables, fruits and whole grains-reduced the rate of fatal myocardial infarction by one-third and the rate of sudden death from cardiac causes by two-thirds. Esposito et al. [110], randomized 180 patients (99 men, 81 women) with the metabolic syndrome to a Mediterranean style diet, characterized with whole grains, vegetables, fruits, nuts, and olive oil versus a cardiac-prudent diet with fat intake <30%. After a follow up of 2 years, subjects in the intervention diet showed greater weight loss, had lower C-reactive protein and proinflammatory cytokine levels, had less insulin resistance, as well as lower total cholesterol and triglycerides and higher HDL cholesterol. The prevalence of metabolic syndrome was reduced to one half. The Japan Public Health Centre-based study [111] showed that eating more w-3 fatty acids by increased intake of fish was associated with significant reduction in cardiovascular disease and cardiac mortality.

## 14. The Indian Experiments of Infarct Survival

 Acute myocardial infarction(AMI) is associated with hyperglycemia, hypertrigly- ceridemia, hyperinsulinemia, increased FFA, free radical stress, IL-6, TNF-alpha, and deficiency of antioxidant vitamins and w-3 fatty acids, which appear to be responsible for complications and deaths among these patients [27, 112, 113, 117–127]. Therefore, AMI or acute coronary syndrome (ACS) appears to be the best model to examine the effects of any intervention on various biochemical markers and associated factors during AMI. Recent studies [28–30], indicate that eating high-fat, refind carbohydrate-rich fast foods (western diet), can produce a similar proinflammatory state in our body, resulting into endothelial dysfunction, which may have adverse effects in patients with AMI. It is therefore logical to avoid western diet in patients with ACS, and administer diet which is beneficial to vascular endothelium and myocardium. There is limited evidence regarding the role of dietary intervention in patients with AMI [112, 113, 117, 118]. The aim of the Indian experiment was to determine the effects of a diet rich in w-3 fatty acids, vitamins, minerals and antioxidants (fruits, vegetables, legumes, walnuts, almonds, fish, mustoard, and soyabean oils) and low in refined carbohydrates, in patients with (AMI) [113, 117–119].

 All patients with a diagnosis of ACS were assigned to an intervention diet (*n* = 204) or a control diet (*n* = 202) within 48 hours of the onset of the symptoms of AMI. The intervention group was advised to consume 600 g/day of fruits, vegetables, legumes, and almonds, and walnuts, in a soup or semisolid form. Tomato soup, skimmed milk, and curd (yogurt) were commonly used to mix crushed almonds and walnuts and other foods, which were grilled with mustered oil. The control group was advised a low-fat diet consistent with National Cholesterol Education Program. Clinical characteristics, time elapsed from symptom onset to the index infarction, site of infarction, drug therapy, and final diagnosis were comparable between the two groups. Intake of foods and selected nutrients was assessed during the 1 week and after 1 year. After 1 week, plasma lipid peroxides, vitamin C, and lactate dehydrogenase levels were determined. Compared with the control groups, patients allocated to the dietary intervention consumed significantly greater amounts of fruit, vegetables, pulses, almonds, walnuts, oils, and fish both during the first trial week [113] and 1 year after AMI [118]. The consumption of *n*-3 fatty acids was also significantly greater in the intervention group than in the control group (1.8 ± 0.66 versus 0.65 ± 0.4 g day^−1^, *P* < .01, Table 2 ). The consumption of proinflammatory foods, such as butter and clarified butter, refined starches and sugar were significantly greater in the control group than in the intervention group (Table 2). Plasma lipid peroxide level decreased significantly in the intervention group compared with the control group, indicating a decrease in oxidative stress which is protective against proinflammatory IL-6 and TNF-alpha as well as endothelial disfunction, although these data were not measured in our study. Lactate dehydrogenase (LDH) level increased less in the intervention group than in the control group, indicating that myocardial damage was prevented by the cardioprotective diet (Table 3). The increased intake of *n*-3 fatty acids from mustard and soy bean oil associated with the Mediterranean diet might be responsible for the significant reduction in the cardiac enzyme LDH and lipid peroxides in the intervention compared with the control group. Total cardiac events, including fatal and non-fatal myocardial infarctions and sudden cardiac deaths, were significantly lower in the intervention group compared with the control group, both after 6 weeks [117] as well as after one year (Table 4) [118]. These Beneficial effects may be due to protection of mitochondria from damageing effects of free radicals.

 The effects of 1 year of treatment with fish oil (122 patients, eicosapentaenoic acid, EPA 1.08 g day^−1^) mustard oil (120 patients, alpha-linolenic acid 2.9 g day^−1^), and no treatment (118 patients, placebo group) on the outcome of patients with suspected AMI were compared in a randomized, placebo-controlled trial) [120]. Treatments were administratered within, on average 18h of onset of symptoms. Clinical characteristics, extent of cardiac damage and rise of cardiac enzymes and lipid peroxides were comparable among the three groups at study entry. After randomization, angina pectoris (18.0% and 21.6% versus 42.3%), arrhythmias (13.1% and 13.3% versus 28.7%) and poor left ventricular function (22.8% and 26.6% versus 47.4%) were significantly lower in the fish oil and mustard oil treatment groups compared with the placebo group. Sudden cardiac deaths (1.6% and 1.6% versus 6.6%), total cardiac deaths (11.4% and 13.3% versus 22.0%) non-fatal infarctions (13.0% and 15.0% versus 25.4%), and total cardiac events (24.5% and 28.2% versus 47.4%) were also significantly lower in the two intervention groups. A modest improvement in dyslipidaemia and a decrease in oxidative damage were observed in the fish oil and mustard oil groups but not in the placebo group. On the third and the fifth day after AMI, serum glutamic oxalotransaminase (SGOT) and LDH cardiac enzymes showed greater decline in the fish oil and mustard oil groups compared with the placebo group. These intervention trials indicate that further studies should be conducted with Columbus diet and Columbus oil (olive oil+lin seed oil) in patients with AMI, to demonstrate, cytokine and endothelial function mediated mechanisms, in the pathophysiology of complications and deaths, among these patients.

 There is no precise and proven guideline for dietary advice, in patients with AMI, which may be protective against recurrent cardiac events. A Columbus soup (tomatoes, grapes, vegetables, walnuts, almonds, lin seed, and olive oil) or yogurt containing, walnuts, almonds, raisins, could be prepared for ready use, for nonpharmacological intervention, among patients of AMI. Such recepies have been commonly used by us in our studies and clinical practice [113, 117–123]. These strategies can influence receptors, enzymes and nitric oxide (NO) secretion via increased intake of w-3 fatty acids [124, 125] which are protective. These methods are similar to dietary approaches proposed in the the DASH diet used in earlier studies [16–18] in patients with multiple risk factors of CAD, indicating metabolic syndrome. The effects of diet on dyslipedimia and glucose were much better when these dietary interventions were combined with exercise [58, 59].

## 15. Recommendations

 It is clear that high doses of EPA and DHA (3 to 4 g/day) have been shown to increase systemic arterial compliance, indicating that marine omega-3 PUFAs improve endothelial function [27, 126–129]. Therefore, a diet containing fish or fish oil supplements or columbus foods (eggs, chicken, and oils) rich in w-3 fatty acids [130], may be advised to provide protection against western diet-induced inflammation. Studies with ALA have been conflicting, with some positive and some negative studies. In a controlled trial [32], Columbus eggs showed beneficial effects on blood lipoproteins. Additionally, the omega-3 PUFAs exhibit an antiinflammatory effect. EPA and DHA reduced tumor necrosis factor, interleukin-6, vascular cellular adhesion molecule-1, and E-selectin at relatively low doses ranging from 0.3 to 1 g/day. Although fewer studies have been performed, the results have been mixed with respect to the effect of ALA on inflammatory markers such as C-reactive protein, vascular cellular adhesion molecule-1, E-selectin, and interleukin-6. The mechanistic studies with EPA and DHA have pointed to an antiarrhythmic effect, and data with ALA are less conclusive. Studies attempting to attribute other antiatherogenic properties to EPA, DHA, and ALA have not been consistent, and many of these studies have demonstrated effects at doses much higher than those used in clinical end point trials. As a result of the previously mentioned omega-3 PUFA trials and recent epidemiologic evidence, the American Heart Association has published guidelines for the consumption of fish and fish oil, indicating that patients with CAD should try to consume a combination of EPA and DHA totaling 1 g/day. Many patients, however; do not enjoy eating fish or have concerns about pollutants. Rich sources of ALA, including canola, lin and flax seed meal, and walnuts, can be easily incorporated into the western diets.

 Columbus oil containing olive oil+lin seed oil could be the best combination along with western diet, for prevention of metabolic syndrome. If clinical trials demonstrate that ALA is as effective as EPA and DHA in reducing cardiovascular events, the public health implications could be significant. The evidence supports a role for fish oil (EPA, DHA) or fish in secondary prevention, because the clinical trials have demonstrated a reduction in total mortality, CHD death, and sudden death. The evidence from these trials has indicated that EPA plus DHA supplementation in the range of 0.5 to 1.8 g/day provides significant benefit. More research is needed to determine whether the benefits of fish oil or fish extend to the world population in secondary and primary prevention.

The data on the plant-based n-3 PUFA, ALA, is very promising. However, the existing studies were small, and a large randomized controlled trial is needed before recommendations can be definitely made for CHD prevention. The data for ALA indicate, possible reductions in sudden death and nonfatal myocardial infarction, suggesting other potential cardioprotective mechanisms other than a predominately antiarrhythmic role. An urgent need exists to perform more clinical trials with ALA because the results of such trials could have significant public health implications. Recommendations are given in Table 6.

 Diet and lifestyle intervention can reduce the risk for conversion of IFG/IGT to type 2 diabetes. Clinical studies, indicate that metformin or thiazolidinediones also reduce risk for type 2 diabetes in people with IFG or IGT. On the other hand, no clinical trial evidence indicates that these drugs will reduce risk for cardiovascular disease events in patients with the metabolic syndrome. Currently, metformin, or thiazolidinediones are not recommended solely for the prevention of diabetes. The cost-effectiveness of this approach has not been established. When patients with type 2 diabetes concomitantly exhibit other features of the metabolic syndrome they are at particularly high risk for cardiovascular disease. Clinical trials show that high priority should be given to treatment of dyslipidaemia and hypertension. Glycaemic control to a haemoglobin A1c of less than 7% will reduce microvascular complications and could decrease risk for macrovascular disease as well. The use of lipid-altering, antihypertensive, and hypoglycaemic drugs can modify insulin sensitivity and bodyweight. Metformin and thiazolidinedi-ones improve insulin sensitivity but have discrepant effects on body weight: metformin reduces weight whereas thiazolidinediones increase it. The increase in weight in patients treated with insulin secretagogues (sulfonylureas and repaglinide or nateglinide) and insulin results mostly from improved glycaemic control and increases in caloric intake as a result of hypoglycaemia. With the exception of nicotinic acid, lipid-altering drugs do not affect insulin sensitivity or weight, whereas the effect of antihypertensive drugs is more complex. Beta-adrenergic blockers and thiazide diuretics might decrease insulin sensitivity but less so at low doses, whereas ACE inhibitors and angiotensin II receptor antagonists have variable effects. By uncertain mechanisms, ACE inhibitors and angiotensin II receptor antagonists seem to decrease the incidence of type 2 diabetes.

 Thrombogenic risk factors are characterised by elevations of fibrinogen, plasminogen activator inhibitor 1, and possibly other coagulation factors. The only available clinical approach to an increased risk for arterial thrombosis in patients with diabetes is low-dose aspirin or other antiplatelet drugs. These drugs are universally recommended unless contraindicated in patients with established cardiovascular disease. Their efficacy in patients with type 2 diabetes in the absence of cardiovascular disease has not been established through clinical trials, although they are widely recommended. In other people with the metabolic syndrome, coenzyme Q10 and aspirin prophylaxis are a therapeutic option when the risk for cardiovascular disease events is judged to be relatively high. A proinflammatory state or premetabolic syndrome is identified by elevated cytokines (e.g., TNF-alpha and interleukin 6) as well as by elevations in acute phase reactants (C-reactive protein and brinogen). An elevated concentration of C-reactive protein is widely thought to be an indicator of a proinfammatory state and to be associated with higher risk for both cardiovascular disease and diabetes. Lifestyle therapies, especially weight reduction, moderate physical activity and alcohol intake in moderation might reduce concentrations of this cytokine and thus can mitigate an underlying inflammatory state. No specific antiinflammatory drugs are available to treat the proinammatory state. However, several drugs used to treat other metabolic risk factors—statins, fibrates, thiazolidinedione, and coenzyme Q10, have been reported to reduce concentrations of C-reactive proteins and cytokines. However, these agents (accept w-3 fatty acids, coenzyme Q10 or a Mediterranean diet or a DASH diet), cannot be recommended specifically to reduce a proinflammatory state independent of other risk factors [125, 131]. The association between overall dietary patterns and mortality due to cardiovascular disease and other chronic diseases is not unknown. In one study [132], a population of >70 000 apparently healthy US women over the course of 18 years, was followed, assessing dietary intake repeatedly. By applying factor analysis, the authors identified 2 major dietary patterns. A greater adherence to the pattern labeled as *prudent* (characterized by a high consumption of plant foods such as vegetables, fruit, legumes, and whole grains as well as fish and poultry) was related to a 28% reduced risk of cardiovascular disease mortality and a 17% reduced risk of premature all-cause mortality. By contrast, a greater adherence to the pattern labeled as *western* (characterized by a high consumption of red and processed meat, refined grains, french fries, and sweets) was associated with a 22% increased risk of cardiovascular disease mortality, a 16% increased risk of cancer mortality, and a 21% increased risk of premature all-cause mortality. The observed associations were independent of known risk factors including age, smoking, physical inactivity, body mass index, and total caloric intake. Nutritional recommendations to prevent chronic diseases and promote longevity may need to focus on overall dietary patterns rather than individual nutrients. This large cohort study further confirms the findings of Lyon heart study and the Indo-Mediterranean diet heart study as well as other views regarding protective effects of Columbus foods in the prevention of CVD and all cause mortality [27, 133]. NCX1.3 is more sensitive to inhibition by ALA than NCX1.1. In addition, only w-3 PUFA inhibits NCX1.1, but several classes of fatty acids inhibit NCX1.3. The differential sensitivity of NCX isoforms to fatty acids may have important implications as therapeutic approaches for hypertension, heart failure, and arrhythmias [134].

## Figures and Tables

**Table 1:** Food and nutrient intake among hunter-gatherer and western population.

Food and nutrient	Hunter-gatherer	Western population
Energy density	Low	High
Protein	High	Low-moderate
Animal	High	Low-moderate
Vegetable	Very low	Low-moderate
Carbohydrate	Low-moderate (slowly absorbed)	Moderate-rapidly absorbed
		
Fiber	High	Low
Fat	Low	High
Animal	Low	High
Vegatable	Very low	High
Total w-3	High (2.3 g/day)	Low (0.2 g/day)
Ratio w-6 : w-3	Low 2.4	High 15-20
Vitamins and minerals	High	low

Modified from Simopoulos 2003 [14].

**Table 2:** Estimated fatty acid consumption in the late Paleolithic period.

Sources	Fatty acids (g/day) en 35.65/day
Plants	
Linoleic acid	4.28
Alpha-linoleic acid	11.40
Animal	
Linoleic acids	4.56
Alpha-linolenic acid	1.21
Total	
Linoleic acid	8.84
Alpha linolenic acid	12.60
Animal	
Arachidonic acid (w-6) (AA)	1.81
Eicosapentaenoic acid (w-3) (EPA)	0.39
Docosatetraenoic acid (w-6) (DTA)	0.12
Docosapentaenoic acid (w-3) (DPA)	0.42
Docosahexaenoic acid (w-3) (DHA)	0.27
Ratios of w-6/w-3	
Linoleic acid/alpha linolenic acid	0.70
AA+DTA/EPA+DPA+DHA	1.79
Total w-6/w-3	0.79

Modified from Eaton et al. in Simopoulos [14].

**Table 3:** Nutrient composition in the late Paleolithic and current recommendations.

Nutrient	Late Paleolithic	Current recommendation
Total dietary energy %		
Protein	33	12
Carbohydrate	46	58
Fat	21	30
Alcohol	−0	moderate alcohol
P/S ratio	1.41	1.00
Cholesterol, mg	520	300
Fiber, g	100–150	30–60
Sodium, mg	690	1100–3300
Calcium, mg	1500–2000	800-1600
Ascorbic acid, mg	440	60

Modified from Eaton et al. in Simopoulos [14].

**Table 4:** Ethnic differences in fatty acid levels in thrombocytes phospholipids and percentage of all deaths from cardiovascular disease.

	Europe and USA %	Japan %	Greenland Eskimos %
Arachidonic acid (20 : 4w6)	26	21	8.3
Eicosapentaenoic acid (20 : 5 w-3)	0.5	1.6	8.0
Ratio of w-6/w-3	50	12	1
Mortality from cardiovascular disease	45	12	7

Modified from Weber by Simopoulos 2003 [14].

**Table 5:** Fatty acid ratio in the diets.

Subjects	w-6/w-3	
Paleolithic	0.79	Estimated
Greece prior to 1960	1.00–2.00	Current 7.10
Japan	4.00	Early 1-2
India, rural	5–6.1	Prior to 1960, 3–4
India urban	38–50	Prior to 1960, 5–10
UK	15.00	Prior to 1960, 10.00
Northern Europe	15.00	Prior to 1960, 10.00
USA	16.74	Prior to 1950 7–8

Modified from Simopoulos 2003 [3, 14].

**Table 6:** Dietary guidelines and desirable level of risk factors for populations.

Factors	Desirable Values
Energy (k calories/day)	1900–2300
Total Carbohydrate (k calories/day)	65.0
Complex Carbohydrate (k calories/day)	55.0
Total Fat (k calories/day)	21.0
Saturated Fatty Acids (k calories/day)	7.0
Polyunsaturated Fatty Acids (k calories/day)	7.0
Polyunsaturated/Saturated Fat Ratio	1.0
*n*-6/*n*-3 Fatty Acid Ratio	1 : 1
Dietary Cholesterol (mg/day)	100
Whole Grains (wheat, rice, corn, and legumes) (g/day)	400–500
Fruit, vegetables, and nuts (g/day)	400–500
Salt (g/day)	<6.0
Brisk Walking (km/day)	9.0
Meditation/pranayam (minutes/day)	30.0

Body Mass Index (kg/m^2^)	
Range	19.0–23.0
Average	21.0

Waist-Hip Girth Ratio	
Male	<0.88
Female	<0.85

Serum Total Cholesterol (mg/dL) (4.42 mmol/L)	<170
Mild Hypercholesterolemia (mg/dL) (4.42–5.20 mmol/L)	170–200
Hypercholesterolemia (mg/dL) (>5.20 mmol/L)	>200
Low Density Lipoprotein Cholesterol (mg/dL) (2.32 mmol/L)	<90
Borderline High (mg/dL) (2.32–2.84 mmol/L)	90–110
High (mg/dL) (2.84 mmol/L)	>110
Triglycerides (mg/dL) (1.7 mmol/L)	<150
High Density Lipoprotein Cholesterol (mg/dL) (0.9 mmol/L)	>40 men, >50 women
Blood Pressure (mmHg)	<135/88
Drug therapy in view of high risk of diabetes and CAD.	Amblodipine, ACE-I, receptor blockers and new beta-blockers? Fish oil, aspirin, and statins

Modified from Indian Consensus Group, J Nutr Environ Med, 1996.
